# Diversity of immune responses in children highly exposed to SARS-CoV-2

**DOI:** 10.3389/fimmu.2023.1105237

**Published:** 2023-03-03

**Authors:** María Úbeda, María del Carmen Maza, Pilar Delgado, Lydia Horndler, David Abia, Laura García-Bermejo, Sergio Serrano-Villar, Cristina Calvo, Ugo Bastolla, Talia Sainz, Manuel Fresno

**Affiliations:** ^1^ Centro de Biología Molecular Severo Ochoa, Consejo Superior de Investigaciones Científicas (CSIC), Universidad Autónoma de Madrid, Madrid, Spain; ^2^ Hospital Universitario Ramón y Cajal, Universidad de Alcalá, IRYCIS, Madrid, Spain; ^3^ Department of Pediatrics, Tropical and Infectious Diseases, Hospital La Paz, and La Paz Research Institute (IdiPAZ), Translational Research Network of Pediatric Infectious Diseases (RITIP), and CIBERINFEC, Madrid, Spain; ^4^ Instituto Sanitario Princesa, Madrid, Spain

**Keywords:** COVID-19, children, immune response, protection, cytokines

## Abstract

**Background:**

Children are less susceptible than adults to symptomatic COVID‐19 infection, but very few studies addressed their underlying cause. Moreover, very few studies analyzed why children highly exposed to the virus remain uninfected.

**Methods:**

We analyzed the serum levels of ACE2, angiotensin II, anti-spike and anti-N antibodies, cytokine profiles, and virus neutralization in a cohort of children at high risk of viral exposure, cohabiting with infected close relatives during the lockdown in Spain.

**Results:**

We analyzed 40 children who were highly exposed to the virus since they lived with severe acute respiratory syndrome coronavirus-2 (SARS-CoV-2)-infected relatives during the lockdown for several months without taking preventive measures. Of those, 26 reported mild or very mild symptoms. The induced immune response to the virus was analyzed 3 months after the household infection. Surprisingly, only 15 children had IgG anti-S (IgG^+^) determined by a sensitive method indicative of a past infection. The rest, negative for IgG anti-N or S in various tests, could be further subdivided, according to IgM antibodies, into those having IgM anti-S and IgM anti-N (IgG^−^IgM^high^) and those having only IgM anti-N (IgG^−^IgM^low^). Interestingly, those two subgroups of children with IgM antibodies have strikingly different patterns of cytokines. The IgM^high^ group had significantly higher IFN-α2 and IFN-γ levels as well as IL-10 and GM-CSF than the IgM^low^ group. In contrast, the IgM^low^ group had low levels of ACE2 in the serum. Both groups have a weaker but significant capacity to neutralize the virus in the serum than the IgG^+^ group. Two children were negative in all immunological antibody tests.

**Conclusions:**

A significant proportion of children highly exposed to SARS-CoV-2 did not develop a classical adaptive immune response, defined by the production of IgG, despite being in close contact with infected relatives. A large proportion of those children show immunological signs compatible with innate immune responses (as secretion of natural antibodies and cytokines), and others displayed very low levels of the viral receptor ACE2 that may have protected them from the virus spreading in the body despite high and constant viral exposure.

## Introduction

Severe acute respiratory syndrome coronavirus-2 (SARS-CoV-2) is the causative agent of coronavirus disease 2019 (COVID-19) that has caused almost 580 million cases worldwide and more than 6.6 million deaths up to November 2022, according to an independent count by Johns Hopkins University (https://www.arcgis.com/apps/dashboards/bda7594740fd40299423467b48e9ecf6). SARS-CoV-2 preferentially infects the respiratory tract causing a potentially fatal disease. SARS-CoV-2 enters human cells *via* the receptor-binding domain (RBD) of its spike (S) protein that interacts with the angiotensin-converting enzyme 2 (ACE2) receptor (1, 2). ACE2 is a membrane-bound enzyme expressed in numerous cell types and tissues such as the lungs, arteries, heart, and intestine. ACE2 catalyzes the cleavage of angiotensin II (AngII) into angiotensin 1-7, regulating the renin–angiotensin–aldosterone system (RAS), playing a critical role in the homeostasis of tissue microcirculation and inflammation (3, 4).

Currently, it is not completely clear how altered ACE2 levels influence SARS-CoV-2 virulence and relevant COVID-19 complications [reviewed in (5)]. On the one hand, ACE2 has lung protective effects by reducing AngII-mediated pulmonary inflammation (6, 7), but reduced ACE2 levels may restrict virus infection (8). Moreover, high levels of serum ACE2 may protect from infection (9–11), acting likely as a decoy.

COVID-19 infection is usually mild in children who have a better outcome than in adults, although the reasons for this are not fully understood (12). Several theories have been proposed to explain this fact (13, 14). One of the first proposed mechanisms is based on differences in the expression and/or affinity of receptors to SARS-CoV-2 between children and adults. In particular, it was suggested that the lower expression of the viral receptor ACE2 in children in nasal epithelium and serum protects them from severe COVID-19 (15, 16).

The first defense against any pathogen is the innate immune response. After virus penetration in the respiratory tract, an innate immune response is activated in which macrophages and dendritic cells recognize the virus releasing inflammatory cytokines (such as TNF, IL‐1b, and IL‐6) and type I interferons (IFNs) (17). So, differences in antiviral IFN production may also account for those sensitivity differences between children and adults (17). SARS-CoV-2 has several strategies to alter IFN production and/or signaling pathways. Moreover, age-associated increases in the production of inflammatory cytokines have been described, implying that children may be less prone to suffer cytokine storm syndrome (12).

Furthermore, children may have a more robust innate immune response to SARS-CoV-2 due to a trained immunity, likely secondary to other viral infections and/or vaccines and allowing the early control of the disease at the site of entry in the respiratory tract (6, 18). Also, there are developmental variations in immune system function with age (12). Moreover, despite the high transmissibility of SARS-CoV-2, there are highly exposed people who have not acquired the infection. Genetic factors and other risk factors can determine the susceptibility of each individual to infection, but those are largely unknown (19).

Our study aimed to provide insight into why children with reported high-risk exposure to the virus cohabiting without protection with infected relatives remained apparently uninfected despite constant contact with the virus. Our results indicate a wide range of immune responses in children highly exposed to SARS-CoV-2.

## Material and methods

### Patients and sample collection

We analyzed data from children recruited at Hospital Universitario La Paz in June 2020, before the SARS-CoV-2 vaccine implementation. They had repeated high-risk exposures to SARS-CoV-2 (specifically, cohabitation with parents with confirmed COVID-19). A total of 40 human sera were obtained at least 8 weeks after the exposure from their relatives. All parents or legal guardians of the children participants provided written consent to participate in the study, which was performed according to the EU guidelines and following the ethical principles of the Declaration of Helsinki. The study was carried out at the Ramón y Cajal University Hospital in Madrid (Spain) and approved by the local Research Ethics Committee (ceic.hrc@salud.madrid.org, approval number 095/20).

### Anti-S flow cytometry immunoassay

Conformational anti-S antibodies were detected by flow cytometry immunoassay (JFCI) to detect IgG and IgA isotypes as described (20, 21). Briefly, Jurkat cells stably expressing the Wuhan S variant (Jurkat-S-GFP) were incubated for 30 min on ice with a 1:50 dilution of children sera in phosphate-buffered saline (PBS), 1% bovine serum albumin (BSA, Sigma-Aldrich, Europe), and 0.02% sodium azide (Sigma-Aldrich). Cells were centrifuged 2 times for 5 min at 500*g*. The cell pellet was finally resuspended in a 1:300 dilution of mouse anti-human IgG1 Fc-PE (Ref.: 9054-09, Southern Biotech) and goat anti-human IgA Fc-Alexa Fluor 647 (Ref.: 2052-31, Southern Biotech). Samples were then washed and analyzed on a FACSCanto II flow cytometer (Becton-Dickinson). Data were analyzed with FlowJo software (BD). The Ig anti-S to GFP mean fluorescent intensity ratio is used as a relative quantitative value as it correlates with the titer and affinity of specific antibodies to S (20, 21). The umbral of positivity was calculated according to the negative control sera for each isotype.

### ELISA

Detection of linear anti-S1/N antibodies was performed by in-home ELISA in 96‐well plates (MaxiSorp Nunc Immuno Plate) coated overnight at 4°C with S1 or N (2 µg/ml) proteins. The coated plates were incubated with the diluted sera for 1 h at room temperature. Plates were blocked with PBS + 0.05% Tween + 1% BSA for 1 h at RT. Bound antibodies were detected by incubation with mouse anti‐human IgG1 or IgM secondary antibody coupled to horseradish peroxidase (HRP; Southern Biotech) diluted 1/6,000 in 1% BSA in PBS, which was then detected using an ABTS substrate solution (Invitrogen). The OD at 415 nm was determined on an iMark microplate reader (Bio‐Rad). The specificity and sensitivity of the assays were controlled using seropositive adults with PCR+, and prepandemic sera were used as negative controls. Prepandemic sera were all below the cutoff levels.

ACE2 and AngII were measured according to the respective manufacturer’s protocol kits (ab235649 Human ACE2 simple step ELISA kit, Abcam; Human Angiotensin II ELISA kit, Reddot Biotech). The OD at 450 nm was determined on a FLUOstar OPTIMA reader (BMG Labtech).

### Multiplex cytokine assay

A bead-based multiplex assay with the main cytokines released in antivirus response was measured according to the manufacturer’s protocol kit (BioLEGENDplex™ Human Anti-virus response panel). The plate was analyzed on a BD FACSCanto II High-Throughput Sampler Option. Data were analyzed using LEGENDplex™ data analysis software.

### Neutralization assay with a pseudotyped virus

Lentiviral supernatants were produced from transfected HEK293T cells as described previously (20). Briefly, lentiviruses were obtained by co-transfecting plasmids pCMV (gag/pol), pHRSIN-GFP, and a truncated S envelope (pCR3.1-St) using the jetPEI transfection reagent (Polyplus-transfection). Viral supernatants were obtained after 48 h post-transfection. Polybrene (8 µg/ml) was added to the viral supernatants before the transduction of ACE2+HEK293T cells. A total of 35-50 × 10^3^ ACE2+HEK293T cells per p48 well were seeded the day before transduction. Diluted plasma (one-fourth) was incubated with viral supernatant for 1 h at 37°C before addition to the cells. Cells were left in culture for 48 h, resuspended in PBS with 2% FBS and 5 mM of EDTA, and fixed with 2% paraformaldehyde. GFP+ cells were then analyzed on a FACSCalibur flow cytometer (Becton-Dickinson). Data were analyzed with FlowJo software (BD).

### Statistical analysis

Significant differences between groups were assessed by the multiple comparisons Kruskal–Wallis test, corrected by controlling the false discovery rate using the Benjamini, Krieger, and Yekutieli test. All data and figures were analyzed and represented using the GraphPad Prism 8 software.

## Results

### Patient stratification

We analyzed a cohort of 40 children, some of them siblings, living at high risk of viral exposure cohabiting in a close and familiar unit with one or two infected relatives (parents with confirmed COVID-19 by PCR) during the lockdown in Spain from March to May 2020 and without taking any preventive measure at home. Since most of the children were asymptomatic or with mild and non-specific symptoms, they did not attend the hospital for analysis until the release of lockdown, and no PCR could be obtained. More than 2 months after the virus risk contact, the serum was collected. All clinical, demographic, and immunological data are reported in **Table S1**. Some children (62.5%) reported mild or very mild symptoms during the high-risk exposure period compatible with a possible infection by SARS-CoV-2. None of the subjects showed signs of any other pathology in the 45-60 days previous to blood collection.

**Figure 1:**
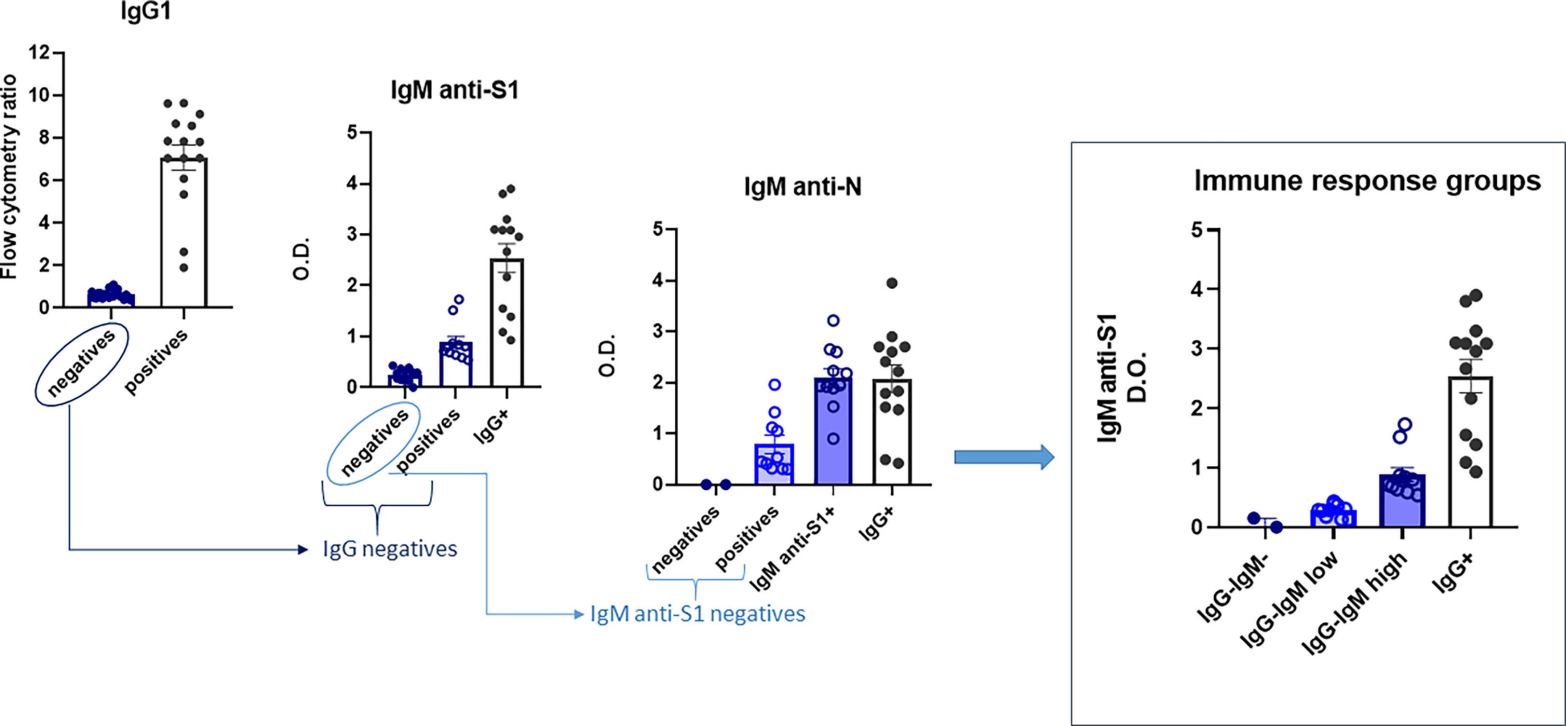
Flowchart of the immunoglobulin profile in children to evaluate their immunological status. Four groups were finally defined: IgG^−^IgM^−^, IgG^−^IgM^low^, IgG^−^IgM^high^, and IgG^+^. Sera were analyzed as described in the *Material and methods*. Results shown are the mean values of one duplicate experiment of the three independent experiments performed with similar qualitative results.

The children’s immunological status was evaluated using various tests (ELISA and flow cytometry) (see **Figure 1** for the flowchart). First, they were tested using a very sensitive assay, more sensitive than routine clinical tests, to measure IgG1 formation to S virus protein by flow cytometry (JFCI) (20, 21) to track past SARS-CoV-2 infection. The Ig anti-S to GFP mean fluorescent intensity ratio is used as a relative quantitative value as it correlates with the titer and affinity of specific antibodies to S. Only 15 children showed detectable antibodies by this assay (**Figure 1**), and eight of them were asymptomatic. Other serological assays, such as conventional ELISA IgG anti-S1 or anti-N, only detected nine (see **Figure S1**).

Next, we performed a more complete serological study evaluating the presence of other isotypes against the same and/or other SARS-CoV-2 epitopes (IgM/IgA anti-S and IgM/IgG anti-N). First, the sera were checked for IgM isotype against S1 and N viral proteins by ELISA. The 15 seropositive children were also positive for IgM anti-S1. Of the 25 IgG anti-S seronegative children, all assays could be performed completely only in 20, allowing a subclassification into those with IgM antibodies against both S1 and N proteins (IgG^−^IgM^high^) and those negatives for IgM anti-S1 but positive for IgM antibodies against N protein (IgG^−^IgM^low^) (**Figure 1**). Only two IgG-seronegative children were also negative for both IgM antibodies. Finally, we analyzed the presence of the IgA isotype against S by the JFCI test. Once again, seropositive IgG anti-S was also positive for IgA anti-S (**Figure S1**), while only nine in the seronegative group were positive in IgA, most of them (*n* = 7) belonging to the IgG^−^IgM^low^ subgroup (**Figure S1**).

Altogether, those assays allow us to subdivide the children according to their immunoglobulin profile into four groups:

1. IgG^+^: children with IgG and IgA anti-S by JFCI, IgM anti-S1 by ELISA, and most also positive for IgG anti-S and anti-N IgG by ELISA (*N* = 15).
2. IgG^−^IgM^high^: children with IgG anti-S and anti-N negative by three independent tests but medium to high levels of IgM anti-S1 and anti-N (*N* = 11). Two of them were also positive for IgA anti-S, determined by JFCI.
3. IgG^−^IgM^low^: children with IgG anti-S and anti-N negative by three independent tests. IgM anti-S1 negative by ELISA but detectable levels of IgM anti-N. Most (70%) were positive for IgA anti-S. One child was eliminated for subsequent analyses due to a previous developmental pathology (*N* = 9).
4. Negative: IgG^−^IgM^−^IgA^−^. Negative in all serological tests (*N* = 2).

Neither association with the type of symptoms nor correlation with sex for those four groups was observed. Group IgG^−^IgM^high^ tends to be younger, with 63.6% of children under 12 years of age, while there were only 40% and 44% in the IgG^+^ and IgG^−^IgM^low^ groups, respectively (**Figure S2**). Of note, the distribution of this immunoglobulin profile was not related to belonging to the same family unit. A total of 22 families participated in the study, 14 of them with two or more children infected. In six of those families (43%), the siblings showed a different pattern of immune response (**Figure S2**).

### Immune and biological responses

**Figure 2:**
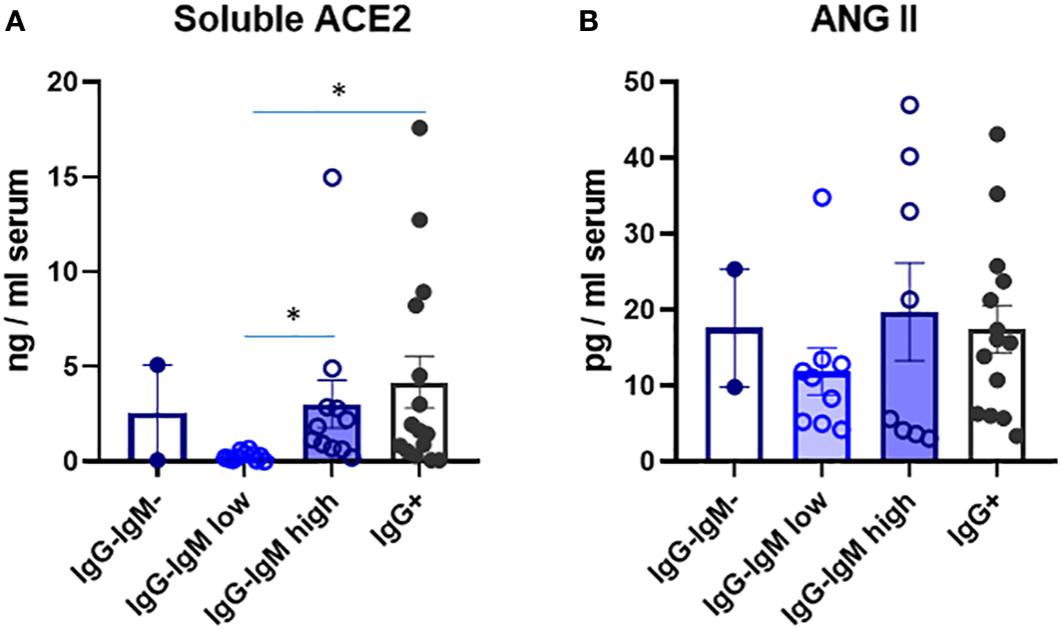
Levels of soluble ACE2 **(A)** and ANG Il **(B)** in serum of children according to their immunological status. Serum levels were analysed by ELISA as described in methods in the indicated four groups: IgG^-^IgM, IgG^-^IgM^low^, IgG^-^IgM^high^, IgG^+^. Results shown are the mean values of one duplicate experiment of the 2 independent performed with similar qualitative results. *p-adjusted<0,05.

We analyzed several immune and biological parameters in those children’s sera looking for clues on their possible innate resistance to infection. ACE2 is a virus receptor, and some reports have attributed the lower susceptibility of children to lower ACE2 levels (13). We indeed detected much lower serum ACE2 levels that we reported for adults in the adult population (9). Very interestingly, the IgG^−^IgM^low^ group had significantly much lower levels of ACE2. No differences among the other groups were observed (**Figure 2**).

**Figure 3:**
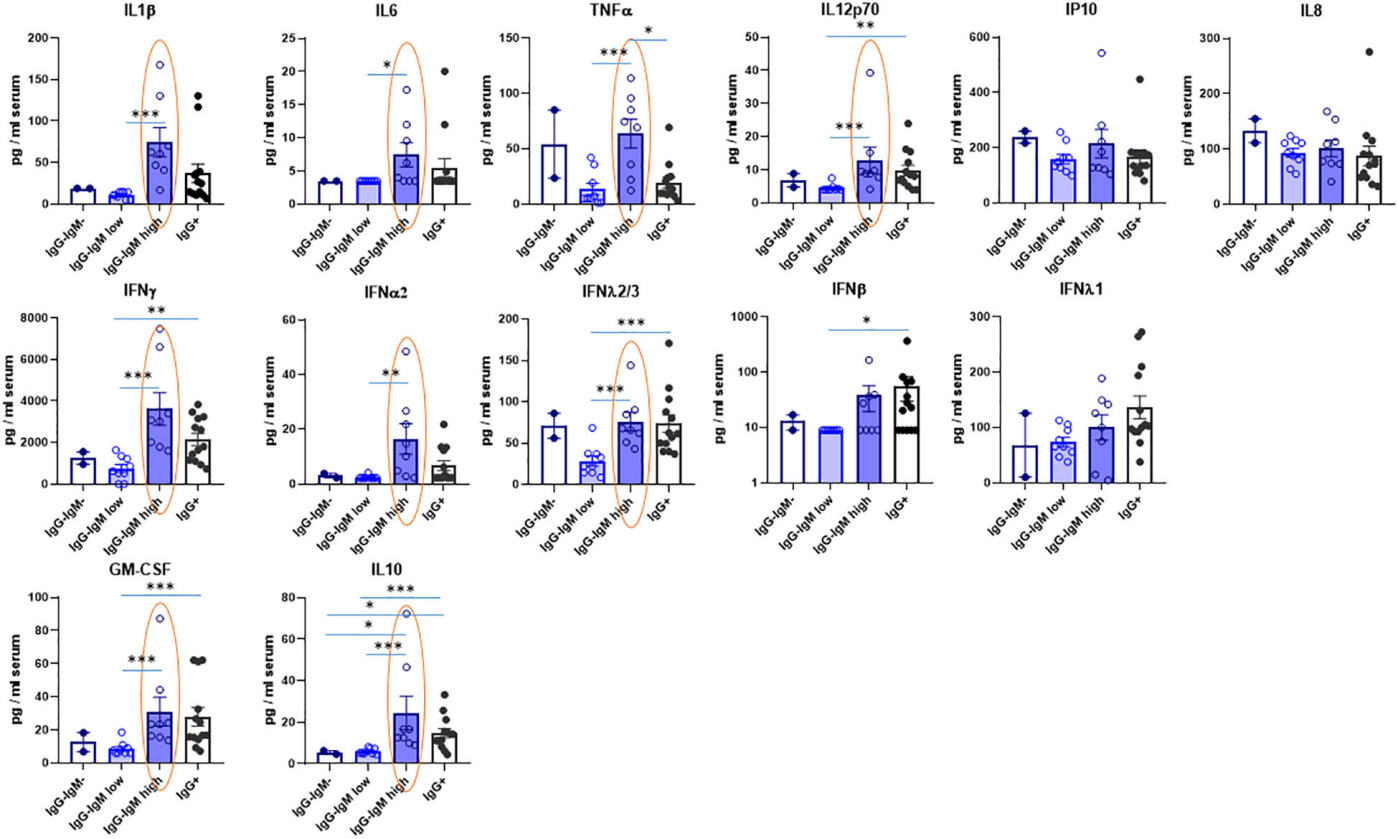
Levels of cytokines in serum of children according to their immunological status. Serum levels were mesured by Multiplex Cytokine Assay as described in methods in the indicated four groups: IgG^-^IgM^-^, IgG^-^IgM^low^, IgG^-^IgM^high^, IgG^+^. The statistical differences between the IgG^-^IgM^low^, IgG^-^IgM^high^ and IgG. groups are indicated. *p-adjusted<0,05; **p-adjusted<0,01; ***p-adjusted<0,005.

Several cytokines were also analyzed in serum to indirectly state the innate immune status (see **Table S1**). Their values considering all children as a single group are within the ranges of those reported for healthy children (i.e., IL-10, GM-CSF, IL-6, TNF) (22). Interestingly, the IgG^−^IgM^high^ group showed significantly higher IL12p70, IL-1, IL-6, and TNF proinflammatory cytokines than the IgG^−^IgM^low^ group (**Figure 3**). Indeed, the IgG^−^IgM^high^ group also had more proinflammatory cytokine TNF than IgG^+^ children. More remarkably, the IgG^−^IgM^high^ group also had the highest levels of several IFNs, such as IFN-α2 and IFN-γ (**Figure 3**). Again, the IgG^−^IgM^low^ group showed the lowest level of IFNs, significantly lower than the other two groups. Moreover, this group showed a very similar pattern of cytokines to the negative group. Once again, a similar pattern was observed in the different groups regarding the levels of IL-10 and GM-CSF (**Figure 3**). Other cytokines such as IL-8 and IP-10 did not significantly differ between the groups.

### The serum of highly exposed IgG-negative children has the capacity to neutralize SARS-CoV-2

**Figure 4:**
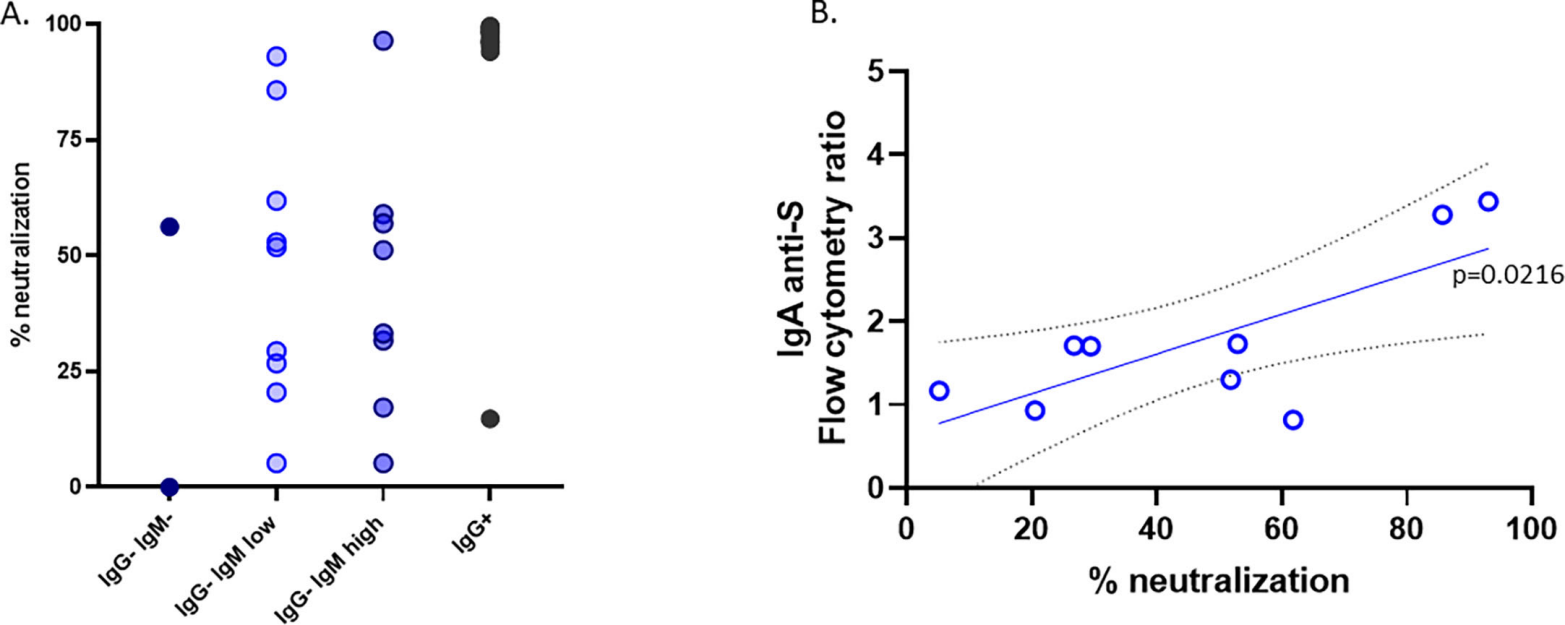
Neutralization. **(A)** The neutralization assay was carried out with the pseudotyped virus as described in the *Material and methods*. **(B)** Group IgG^−^IgM^low^ showed a significant correlation between the neutralizing activity and the levels of IgA anti-S in the serum.

To further explore why subjects in contact with the virus remained IgG seronegative, we investigated the ability of the lentivirus pseudotyped with the spike protein to infect ACE2-expressing cells in the presence of the sera of these subjects. As expected, all but one serum from IgG^+^ children neutralized the virus infection very efficiently (**Figure 4**). Nonetheless, despite having no IgG antibodies against the S protein, most of the sera from the IgG^−^IgM^high^ or IgG^−^IgM^low^ groups showed a broad pattern of neutralizing activity (**Figure 4**). The sera from unexposed seronegative adults or prepandemic children did not neutralize [(9) and data not shown]. Considering that those groups had IgA anti-S, we performed a linear correlation. We found that in the IgG^−^IgM^low^ group, the neutralization of those sera significantly correlates with IgA anti-S1 (*p* = 0.0216) (**Figure 4**).

## Discussion

Children are less susceptible than adults to symptomatic COVID‐19 infection, but the basis for this outcome is unclear and very few studies addressed their underlying cause. Moreover, very few studies analyzed children highly exposed to the virus, who nevertheless remain apparently uninfected. Few studies, much less than in adults, have investigated the response to children (12, 13, 23, 24) but none, to our knowledge, of children cohabiting in close contact with infected patients without taking any preventive measures. Here, we have addressed this question through this descriptive, cross-sectional, and retrospective study.

Most children during the first waves were reported to be infected by close relatives (24). Surprisingly, we found that only 15/40 children in close contact with infected relatives have detectable IgG antibodies in their sera by the very sensitive JFCI test (20) indicative of a previous SARS-CoV-2 infection. Moreover, other ELISA tests against S1 or N also failed to detect IgG against the virus in those IgG-negative (by JFCI) patients. Thus, 25/40 children did not show positivity in any of those three tests despite cohabiting with an infected person(s) for a long time without taking preventive measures.

Nonetheless, a thorough analysis of immune response to track the possible SARS-CoV-2 infection indicated that all but two must have been in contact with the virus and mounted an immune response against it, since they had variable levels of IgM anti-S1 and/or anti-N protein and specific IgA antibodies against the spike protein in their serum. The IgG^−^IgM^low^ subgroup was mainly positive for IgM antibodies against the N protein. Those antibodies may have arisen due to a possible cross-reaction with the common nucleocapsid protein expressed by other coronaviruses. However, nine children in the IgG-seronegative group were positive for IgA anti-S, and most of them (*n* = 7) belong to the IgG^−^IgM^low^ subgroup, indicative of some kind of SARS-CoV-2 infection beyond a possible cross-reaction with another coronavirus.

Since most of the analysis of the epidemiological surveys used IgG anti-SARS-CoV-2 for defining people that pass the infection, extrapolating our data would indicate that the extent of the disease in children would be greater than reported. In this regard, whether COVID-19 is less common in children is debated. Some propose that children are less susceptible, whereas others believe that children become infected as adults but are less likely to be symptomatic (25).

More importantly, our analyses defined two different IgG^−^ seropositive groups according to the levels of IgM, anti-S, or anti-N: IgG^−^IgM^high^, a combination of medium to high levels of IgM anti-S and anti-N, and IgG^−^IgM^low^, only with IgM anti-N. Although those differences were somewhat arbitrary, the subsequent analysis demonstrated that both groups defined very different immune responses to the virus. Thus, the IgG^−^IgM^high^ group, which has higher levels of IgM anti-S1 and anti-N than the IgG^−^IgM^low^ group, also tended to be younger. By contrast, the levels of IgA anti-S, determined by a sensitive flow cytometry test, were more frequent and higher in the IgG^−^IgM^low^ group, although lower than in the IgG^+^ group.

Nonetheless, in many of the other parameters analyzed, those groups have strikingly different behavior suggesting a different immunological/biological response to the virus. Thus, the IgG^−^IgM^low^ group has much lower serum ACE2 than the rest. Furthermore, an analysis of parameters indicative of potential innate responses such as the levels of serum cytokines also revealed striking differences between the groups. The IgG^−^IgM^high^ group has the highest levels of some antiviral IFNs, such as IFN-α2, IFN-λ2/3, and IFN-γ, among all groups, whereas the IgG^−^IgM^low^ group has lower levels. Other cytokines, such as the proinflammatory IL-1/TNF/IL-6, as well as GM-CSF and IL-10, were also higher in the IgG^−^IgM^high^ group than in the IgG^−^IgM^low^ group. Moreover, the levels of those cytokines in the IgG^−^IgM^high^ group were even higher in many instances than in the IgG^+^ group.

Surprisingly, the sera from the IgG^−^IgM^low^ group, despite having lower antiviral and macrophage-activating cytokines and no detectable IgM anti-S, have a weak but detectable viral neutralization ability. Despite not having IgG anti-S, the IgG^−^IgM^high^ and IgG^−^IgM^low^ groups had a similar neutralization capability. Indeed, in the IgG^−^IgM^low^ group, this weak neutralization correlates to IgA anti-S levels.

We do not know the reason for the differences between the two IgG^-^IgM^+^ groups, but we speculate that the IgG^−^IgM^low^ group represents a group of children with the lowest SARS-CoV-2 receptor, ACE2, which may be the reason for the reduced virus entry. The role of ACE2 expression levels on SARS-CoV-2 infection severity is still debated. Membrane-bound ACE2 is the main cellular receptor of SARS-CoV-2, and lower levels are expected to reduce infection of airway epithelial cells. In this regard, it was proposed that the lower expression of the viral receptor ACE2 in children in the nasal epithelium and serum protects them from severe COVID-19 (15, 16) and that patients with a lower expression level of ACE2 are less vulnerable to developing severe symptoms (26). Moreover, sACE2 tends to increase with age (27, 28). The presence of IgA antibodies in mucosal sites against SAR-CoV-2 with good neutralizing activity was described in IgG-seronegative patients with mild disease (29). IgA is the main immunoglobulin in the respiratory tract and contributes to virus neutralization more heavily than IgG (30, 31). Thus, neutralizing IgA anti-S antibodies in the IgG^+^IgM^low^ group could also, besides low ACE2 levels, explain their resistance to a more productive infection.

On the other hand, we propose that the IgG^−^IgM^high^ group could include a group of children with an innate immune response. This hypothesis is based on cytokine levels, which are elevated in this group, having the highest levels of antiviral interferons, such as IFN-α2 and IFN-γ, among all the groups. The protective antiviral response in circulating immune cells of adult COVID patients is strongly associated with a specific subset of IFNs, most prominently IFN-α2 and IFN-γ, which are inversely correlated with severity (32). Interestingly, those are the ones we show elevated in IgG^−^IgM^high^ children. Moreover, children have higher nasal IFN-α2, IFN-γ, and IL-1β cytokine levels than adults. Those cytokines had been associated with lower susceptibility, indicating a stronger mucosal innate immune response in children than in adults that may contribute to milder clinical outcomes (33).

It has been proposed that polyclonal native IgM may protect children from SARS-CoV-2 infection (14). This polyclonal IgM is abundantly present in neonates and children and can recognize viral particles or infected cells, being also able to recognize self- and altered self-antigens. Native IgM may play a role in defense against SARS-CoV-2 since it may neutralize the virus through the recognition of endogenous “danger signals” encoded by the virus (14). The IgM antibodies in the IgG^−^IgM^high^ group may also be natural antibodies and, together with the low ACE2 levels, one of the causes of viral resistance. Aside from IgM, natural IgA production has also been described to be derived from B1 lymphocytes by a T-independent mechanism (30). In this regard, purified IgM and IgA fractions of patient sera display neutralizing activities (34). Unfortunately, we do not have data on T-cell response. Nonetheless, the fact that most of those children, despite being highly exposed, did not suffer a “classical” SARS-CoV-2 infection (defined by the generation of IgG anti-S or anti-N due to antigen presentation and T–B cooperation) may argue against a SARS-CoV-2-specific T-cell response in the IgG-seronegative children. Thus, a rather plausible scenario is that those IgA and IgM antibodies present in IgG-negative children may be natural antibodies derived from B1 lymphocytes by a T-independent mechanism. It is important to note that blood samples were obtained 2 months after the virus exposure, time enough for the IgG isotype switch to occur. B1 cells appear early in the ontogeny, decreasing with age and providing an essential link between innate and adaptive immune systems (35). Our hypothesis is supported by the fact that B1 lymphocytes have been described to be high in children and protect against respiratory viruses such as influenza (36), and it has also been proposed as a mechanism for resistance to SARS-CoV-2 (30, 37). Indeed, B1 lymphocytes secrete natural IgM, IL-10, and GM-CSF (37), and we found higher IL-10 and GM-CSF in the IgG^−^IgM^high^ group, strongly supporting that there are B1 cells involved in the resistance of those children. Unfortunately, we do not have the cellular sample to corroborate this hypothesis.

In summary, we have identified different mechanisms that may play some role in children’s resistance to SARS-CoV-2 infection. Children with higher levels of IFNs in the airways can clear the virus faster as well as children with low levels of ACE2 receptors in which the virus does not have the opportunity to replicate to a high level in the mucosa. Both mechanisms may have prevented the spread of the virus into the body, avoiding a classical T/B response defined by the production of IgG immunoglobulin.

## Data availability statement

The original contributions presented in the study are included in the article/**Supplementary Material**. Further inquiries can be directed to the corresponding authors.

## Ethics statement

The studies involving human participants were reviewed and approved by the local Research Ethics Committee (ceic.hrc@salud.madrid.org, approval number 095/20). Written informed consent to participate in this study was provided by the participants’ legal guardian/next of kin.

## Author contributions

MU, MM, PD, and LH conducted the experiments. MU acquired and analyzed the data. LG-B, CC, and TS provided human biospecimens. MU and MF analyzed and interpreted the data. TS conceived the clinical recruitment of the patients. DA and UB performed bioinformatics to analyze the data. MF and UB conceived and designed this study. MF wrote the manuscript. All authors contributed to the article and approved the submitted version.
