# Supplementary figures and images for: Diversity of immune responses in children highly exposed to SARS-CoV-2
Source: Front Immunol. 2023 Mar 3;14:1105237. doi: 10.3389/fimmu.2023.1105237 (PMC10020361; doi:10.3389/fimmu.2023.1105237)

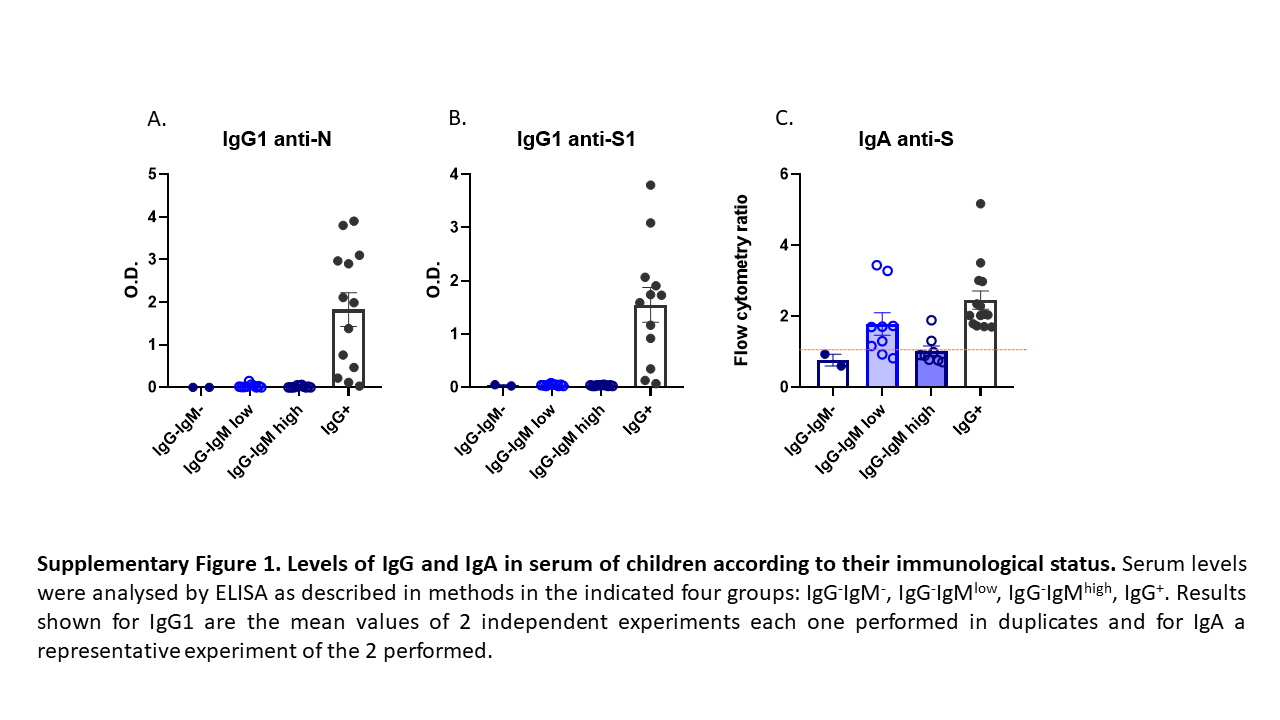

Supplement: Supplementary file 1 [file Image_1.tif]

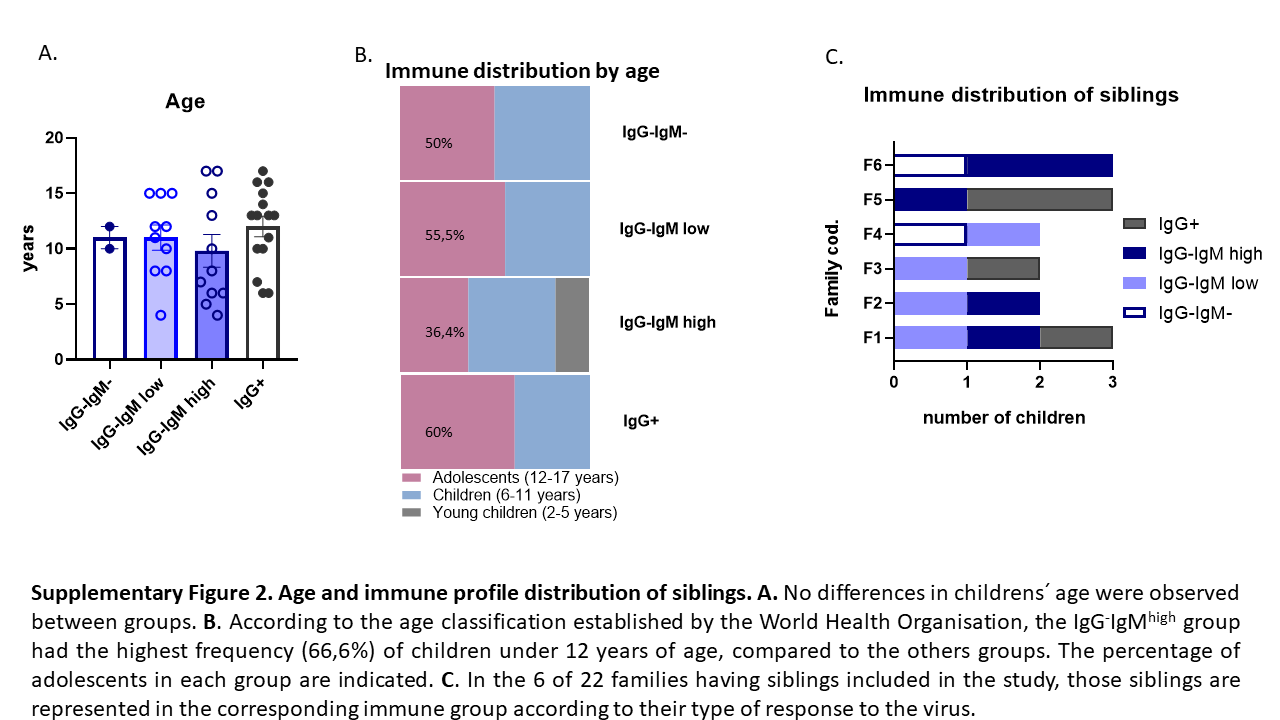

Supplement: Supplementary file 2 [file Image_2.tif]
