# Supplementary material for: Diversity of immune responses in children highly exposed to SARS-CoV-2
Source: Front Immunol. 2023 Mar 3;14:1105237. doi: 10.3389/fimmu.2023.1105237 (PMC10020361; doi:10.3389/fimmu.2023.1105237)
Supplement: Supplementary file 3 [file Table_1.pdf]

				anti-Spike Ab				anti-N Ab				Cytokines													
				JFCI (ratio)		ELISA (O.D.)		ELISA (O.D.)				Multiplex Assay													
Cod	Age	Sex	Symptoms	IgG1	IgA	IgG1	IgM	IgG1	IgM	ACE2 (ng/ml)	AngII (pg/ml)	IL1beta (pg/ml)	IL6 (pg/ml)	TNFalpha (pg/ml)	IP10 (pg/ml)	IFNlambda1 (pg/ml)	IL8 (pg/ml)	IL12p70 (pg/ml)	IFNalpha2 (pg/ml)	IFNlambda2/3 (pg/ml)	GM-CSF (pg/ml)	IFNbeta (pg/ml)	IL10 (pg/ml)	IFNgamma (pg/ml)	neutralization %
75	9	F	URI + F	0,55	0,83					0,37	36,32	56,68	3,50	35,78	137,46	75,09	231,80	7,00	2,30	50,16	20,33	9,00	7,29	1091,37	58,38
76	13	F	URI	0,56	0,90					0,28	14,16	5,00	3,50	20,57	110,82	204,76	132,39	13,79	6,29	96,33	32,65	63,02	21,27	2031,71	43,40
77	6	F	URI	7,05	1,73	1,17	3,09	1,38	1,83	8,94	10,73	116,88	20,08	23,49	181,88	264,78	89,68	16,21	21,84	170,94	61,19	22,94	25,74	3838,78	95,09
78	6	M	URI + F	7,81	5,17	1,74	1,09	2,11	0,42	1,94	6,00	14,82	3,50	24,97	80,56	37,99	275,99	4,00	2,30	36,87	9,32	9,00	11,03	731,48	94,00
80	8	M	URI	0,89	3,44	0,05	0,29	0,07	0,30	0,58	8,32	8,78	3,50	19,13	128,47	37,99	54,85	4,90	2,30	32,67	6,00	9,00	5,00	864,02	93,00
81	10	F	URI	9,62	2,03	3,80	3,09	3,90	2,60	0,87	17,35	39,01	3,50	9,75	173,55	272,53	35,94	23,98	6,22	103,26	62,23	82,05	33,27	3248,54	99,37
82	5	M	URI	0,67	1,31	0,04	1,52	0,00	2,23	2,79	3,00	46,92	3,50	85,04	127,60	149,48	108,70	9,52	10,13	90,17	15,63	39,07	12,73	2018,38	17,20
83	6	F	A	0,49	0,71	0,04	0,64	0,00	0,90	2,86	4,10	41,00	12,01	22,02	543,29	14,83	113,02	9,79	4,94	43,07	23,52	9,00	16,75	3073,74	5,20
84	4	F	URI	0,62	0,89	0,05	0,31	0,02	0,31	17,59	5,55	68,16	14,58	86,82	342,77	95,06	119,36	43,33	25,69	135,41	114,59	9,00	86,22	4997,57	43,64
85	6	F	URI	0,37	0,89	0,05	0,87	0,05	1,93	0,63	3,62	167,67	4,01	74,46	104,13	75,09	74,11	8,90	2,88	67,69	24,40	27,36	12,73	1602,19	58,96
86	10	M	A	0,62	0,93	0,05	0,13	0,01	0,45	0,14	11,05	5,00	3,50	1,00	256,43	112,97	92,53	4,37	2,30	21,94	6,00	9,00	5,85	630,70	20,52
87	8	F	URI	0,46	1,17	0,03	0,43	0,00	1,96	0,66	12,81	5,00	3,50	5,08	228,36	46,78	81,17	4,00	2,30	21,94	6,00	9,00	8,23	1222,38	5,20
88	10		A	1,90						0,06															
89	11	F	URI	8,57	3,01	2,07	2,17	2,97	3,95	0,28	16,12	6,80	3,50	3,03	134,07	102,70	56,65	5,06	3,38	57,09	16,71	9,00	14,48	1439,74	98,45
90	13	F	URI	7,85	2,98	1,91	2,96	3,10	2,70	1,44	6,27	14,82	3,50	13,62	448,49	92,53	48,79	4,00	2,30	61,47	15,63	36,47	15,16	3466,74	99,15
91	13	M	URI	0,54	0,78	0,03	0,80	0,02	3,22	0,92	47,00	130,47	17,27	95,75	185,08	100,14	153,68	15,23	21,84	84,79	44,26	36,47	46,66	3398,19	96,39
92	7		G			0,04	1,73	0,00	2,60	2,20															
93	15	F	URI + F	1,10	1,30	0,08	0,39	0,15	1,12	0,34	5,21	14,82	3,50	5,08	163,44	65,39	110,57	4,00	2,30	14,25	7,72	9,00	5,33	523,05	51,80
94	15	F	URI	0,98	1,70	0,05	0,13	0,03	0,32	0,07	34,82	14,82	3,50	5,08	163,44	65,39	110,57	4,00	2,30	14,25	7,72	9,00	5,33	523,05	29,40
95	10		F + G			0,04	0,69	0,01	1,53	4,90															
96	11	F	URI + F + C	0,56	1,73	0,04	0,37	0,00	1,42	0,00	4,18	14,82	3,50	42,21	99,23	95,06	115,76	4,00	4,16	37,39	11,00	9,00	7,66	1645,44	52,89
98	12	F	URI	0,69	0,82	0,03	0,18	0,02	0,41	0,19	13,48	10,78	3,50	7,33	148,57	105,25	123,55	4,00	2,30	68,64	9,09	9,00	5,00	1348,22	61,80
99	15		URI			0,04	0,72	0,06	2,18	14,98															
100	7	F	A	7,84	1,79	1,73	1,39	2,90	2,22	3,01	21,27	24,94	3,50	8,52	107,02	97,60	124,28	6,84	2,30	39,99	24,70	9,00	11,24	1800,66	98,58
101	12	M	A	0,76	3,28	0,04	0,29	0,01	1,05	0,20	11,90	14,82	3,50	1,00	113,62	55,93	63,87	4,00	2,30	8,50	6,00	9,00	4,51	0,50	85,69
102	13	M	A	7,04	2,35	0,92	3,80	1,99	1,52	0,06	35,25	12,80	3,50	35,78	180,09	105,25	79,47	6,26	2,30	50,16	7,28	20,35	6,20	1645,44	95,98
103	12	M	A	0,48	0,60	0,03	0,15	0,00	0,00	0,06	25,35	18,87	3,50	85,04	260,15	10,10	154,70	4,83	2,30	86,15	6,84	9,00	4,67	953,95	0,00
104	15	M	A	0,39	1,71	0,03	0,28	0,04	0,53	0,05	4,99	10,78	3,50	35,78	133,65	77,55	87,55	7,51	2,30	36,87	18,64	9,00	7,47	0,50	26,73
105	14	F	A	7,04	3,51	0,07	2,67	0,12	2,70	4,53	3,35	130,47	4,93	69,23	178,29	131,14	70,45	9,43	13,57	87,94	32,65	367,73	8,23	2618,17	97,93
106	16	F	F	6,08	2,06					17,59	13,84														96,08
107	13	M	A	9,64	2,04		1,55	0,76	1,47	0,53	43,13	35,01	3,50	14,96	183,67	194,24	90,82	8,11	13,47	116,75	26,80	66,20	14,26	3155,62	96,92
108	17	F	URI + F	0,51	1,89	0,03	0,79	0,00	1,89	1,83	21,34	62,45	9,58	35,78	121,84	4,50	40,91	7,77	14,75	65,79	23,52	165,44	9,01	1786,75	31,65
109	15	F	A	2,64	1,70	0,13	0,93	0,03	2,41	0,83	25,75	37,01	12,01	20,57	109,40	210,02	115,04	15,52	12,32	80,26	62,23	27,94	21,27	2727,98	14,80
110	17	M	URI	0,66	0,91	0,04	0,59	0,02	1,95	0,20	40,21	70,05	6,26	113,80	128,03	188,97	77,92	39,26	48,50	144,05	87,33	9,00	72,29	6610,62	33,20
111	4	M	A	0,97	0,76	0,03	0,84	0,04	2,65	0,69	5,59	16,84	3,50	12,30	281,29	141,60	73,12	8,81	2,30	61,95	13,78	9,00	16,75	3014,91	51,20
112	10	F	F	0,45	0,94	0,05	0,00	0,00	0,00	5,09	9,83	18,87	3,50	23,49	217,01	125,93	111,15	8,90	3,85	56,11	18,36	16,93	6,38	1558,51	56,20
113	8	F	URI	0,70	0,99	0,03	0,54	0,00	1,92	1,16	32,98	60,53	3,50	69,23	222,10	125,93	167,92	4,14	26,73	51,16	16,44	9,00	10,01	7493,01	56,94
115	16	F	A	9,12	1,71	3,09	3,10	3,80	2,90	12,74	15,65	10,78	3,50	9,75	134,93	72,65	32,49	7,26	2,30	62,43	16,98	68,62	11,66	936,24	99,52
116	17	F	A	5,35	2,03	0,35	3,90	0,22	1,79	8,22	5,69	10,78	3,50	7,33	135,77	100,14	48,25	8,55	2,30	49,66	14,30	9,00	4,51	1108,07	98,03
117	13	F	F	8,67	2,29	1,59	3,30	0,47	0,49	1,63	23,75	26,97	3,50	11,01	124,51	92,53	72,98	12,27	2,30	39,99	15,90	9,00	15,61	1157,59	96,16

URI: upper respiratory infection; F: fever; A: asymptomatic; G: gastrointestinal; C: cutaneous
